# Self-rated language proficiency as a context-dependent judgment

**DOI:** 10.3758/s13423-026-02938-z

**Published:** 2026-06-01

**Authors:** Dorit Segal, Anne Neveu

**Affiliations:** 1 Department of Education and Psychology, The Open University, 1 University Road, P.O. Box 808, 4353701 Ra’anana, Israel; 2 Department of Communication Sciences and Disorders, Kean University, Union, NJ USA

**Keywords:** Bilingualism, Foreign language, Self-evaluation of proficiency

## Abstract

While self-ratings of proficiency are widely used in bilingualism research and clinical settings, previous work has found that they often lack reliability within and across bilinguals, across languages, tasks and over time. Yet little is known about the potential effect of conducting these ratings early versus late in a testing session, and specifically, before or after objective tasks of proficiency. We examine this question in a sample of unbalanced Hebrew-English bilinguals (*N *= 186), who completed self-ratings of proficiency in their two languages both before and after they completed a multiple-choice vocabulary test in one of their languages. Importantly, we manipulated the difficulty of the task (easy vs. difficult words) to further probe the role of context in self-judgment of proficiency. Findings revealed that self-ratings were influenced by recent experience: regardless of language, when the vocabulary task was difficult, self-ratings in the language of the task lowered. Moreover, when the difficult task was in the native language, the self-ratings in the second language also decreased, even though the second language was not tested, and post- rather than pre-task self-ratings tended to be more strongly correlated with vocabulary scores. Overall, these findings show that judgments of self-proficiency are dynamic, context-dependent inferences shaped both by prior beliefs of proficiency and immediate experience, underscoring the need to consider individual and task factors when collecting self-reported measures.

**Supplementary Information:**

The online version contains supplementary material available at 10.3758/s13423-026-02938-z.

## Introduction

Self-ratings of language proficiency are among the most widely used measures in bilingualism research and clinical settings due to their low cost, time efficiency, and indicative nature (Hulstijn, 2012; Li et al., 2006). However, previous work has found self-ratings to lack reliability across groups of bilinguals, but also within bilinguals, across languages, against various objective tasks, and over time. Here, we focus on other factors that can affect self-ratings: the timing of their administration within the experimental session and the adjacent completion of an objective test.

When self-rating their proficiency, bilinguals are asked to evaluate their speaking, understanding, reading, and writing skills in each of their languages on a 1 (very poor) to 7 (like a native speaker) scale or a 1 to 10 scale. These ratings have often shown robust correlations with objective proficiency measures, indicating that they provide meaningful estimates of actual language ability. However, these correlations are far from perfect, and their validity has often been questioned, as they vary across and within individuals, and across languages and tasks. For example, Tomoschuk et al. (2019) examined to what extent self-ratings of English proficiency (1–7 scale) predicted scores on an objective proficiency measure (picture naming) in Chinese-English and Spanish-English bilinguals. They found that the relationship between self-ratings and objective proficiency differed across groups and across the scale. At lower self-ratings (e.g., 4 out of 7), Chinese-English bilinguals performed worse on the naming task than Spanish-English bilinguals but at the high end of the scale (e.g., 7 out of 7), Chinese-English bilinguals performed better than Spanish-English bilinguals. This suggests group-specific differences in standards for estimating self-proficiency (see Strong-Krause, 2000, for similar findings). Other studies found differences in the self-estimation of proficiency between different age and proficiency level groups (Kaderavek et al., 2004; Kavé & Halamish, 2015; Ma & Winke, 2019). For example, Kavé and Halamish (2015) found that older adults were more accurate in their self-evaluation of vocabulary knowledge than middle-aged and younger adults. Ma and Winke (2019) asked novice, intermediate, and advanced learners of Chinese to rate their ability to complete spoken Chinese tasks and compared these ratings to actual performance. Novice and advanced learners were more accurate than intermediate learners.

Other studies have shown that the accuracy of self-ratings vary even within the same individuals, across languages, skills, and tasks. For instance, Gollan et al. (2012) measured the correlations between young adult Spanish-English bilinguals’ self-ratings of proficiency (1–10 scale) and their actual performance on language tasks and found significant correlations ranging from *r* =.28 to.50 in English, the dominant language, and from *r* =.43 to.52 in Spanish, the non-dominant language. Similarly, Marian et al. (2007) used factor analysis and reported moderate to strong associations between self-reported proficiency and various objective measures (e.g., reading fluency and grammaticality judgment) in Spanish-English bilinguals. These associations were stronger for the second language (L2) than for the native language (L1), and they differed across skills and tasks (see also Delgado et al. (1999) and Ross (1998) for similar findings). Together, these findings suggest that self-ratings of proficiency are not fixed measures even within the same individuals, but rather, they are context-dependent, probably reflecting both more stable internal standards and external influences: the tools and abilities evaluated.

This view aligns with metacognitive theories showing that people lack access to their actual knowledge, and instead infer it based on various cues, such as beliefs and momentary experiences (Ackerman, 2019; Koriat, 1997). Although individuals strive to maintain stable evaluations of their abilities, current experiences can trigger re-evaluation (Epstein, 1973; Freund & Kasten, 2012). In the context of language, for instance, individuals may hold a general belief about their proficiency, based on accumulated past experience (e.g., “I am good at my L1 and poor at my L2”), yet a recent experience (e.g., experiencing success on a difficult task in the L2) may prompt them to revise this belief (e.g., “maybe I am not as bad as I thought in my L2”). Such revisions may either increase or decrease the accuracy of self-judgment, and their effect may differ for the L1 versus the L2. Indeed, several studies have shown that the self-evaluation of proficiency changes over time with accumulated experience (Chen, 2008; Dolosic et al., 2016). Dolosic and colleagues (2016) asked 24 students aged 14**–**18 years, with English as L1, to evaluate their French proficiency before and after enrolling at a French summer camp immersion program, and compared their evaluations to their actual proficiency. The accuracy of evaluations improved with time, demonstrating that experience changes self-evaluation of proficiency. However, it is not clear whether the change in evaluation was due to improvement in proficiency, evaluation ability, or both.

Importantly, if self-evaluation of proficiency is sensitive to both beliefs and recent experience, then participation in an experimental task may influence how individuals evaluate their own abilities, and this influence can differ across languages. Indeed, Hirosh and Degani (2021) randomly assigned Hebrew-English bilinguals to two groups matched on objective measures of proficiency. Participants in both groups performed experimental tasks either in Hebrew (L1) or in English (L2), and then provided self-reports of proficiency and language use. The results showed that participants reported greater use of the language in which they had performed the task. Although the underlying mechanism was not directly tested, the authors hypothesized that this effect reflects the influence of the experimental session on self-reports. Delgado et al. (1999) asked relatively balanced Spanish-English bilinguals to rate their skills in each language before and after completing an objective proficiency test(the Woodcock-Muñoz Language Survey). Post-task ratings were lower than pre-task ratings and were slightly more accurate, but only for Spanish. These findings support the assumption that experience influences the evaluation of self-proficiency, that this influence differs across languages, and that the timing of self-ratings within experimental sessions can affect their accuracy. However, because the participants in that study were relatively balanced bilinguals and the task had a fixed level of difficulty, it remains unclear how differences in language dominance and task difficulty interact to shape self-judgments of proficiency.

In the current study, we aimed to directly test the effects of language dominance and task difficulty on self-rating of proficiency by asking unbalanced bilinguals to complete both easy and difficult tasks in both their languages.

## Method

### Transparency and openness

We report how we determined our sample sizes, all data exclusions, all manipulations, and all materials. Data, analysis code, and research materials are available at: https://osf.io/vk5pt/overview?view_only=d4a3dd220551483e8173be1ccdd29911. Power analysis was conducted in R (WebPower package; Zhang & Yuan, 2018), and the study received ethics approval (3681).

### Participants

A repeated-measures ANOVA power analysis, with four groups, four measurements (pre- and post-task and in Hebrew and English) per participants, an effect size of 0.3, alpha set at.05, and power set at.8 showed that a sample size of *N* = 176 (*N* = 44 per group) would be needed to detect an interaction between group, time, and language. To account for potential noise in online testing, we aimed to recruit approximately 45 participants per group.

Two hundred and eight participants were originally recruited for the study. Except for seven participants who took part for course credit, all others were recruited through an Israeli survey company (Panel4all) and were compensated for their participation. Participants whose questionnaire completion time exceeded three standard deviations from the group’s mean completion time (*N* = 3) or who reported using a dictionary during the task (*N* = 14) were excluded. All remaining participants were born in Israel. However, five participants who reported acquiring Hebrew after the age of 4 years were also excluded. The cutoff at age 4 was determined based on the Israeli context, where formal education typically begins at ages 3**–**4 years, and most children are already immersed in Hebrew by this stage. Participants reporting later acquisition either misinterpreted the question (e.g., referring to formal instruction rather than initial exposure) or were raised in a non-Hebrew-speaking environment during early childhood. In contrast, exposure to English in Israel typically begins later. Children usually start learning English in school around ages 8**–**9 years and continue through high school and into higher education. Although English is not used for daily communication in most contexts, Israelis are regularly exposed to English through academic use, media consumption (e.g., music and movies), and engagement with digital and social platforms.^1^

The final sample consisted of 186 participants, 95 identified as male, 90 as female, and one preferred not to answer the question. These participants were randomly assigned to one of four groups, in which they completed either a Hebrew vocabulary task with “easy words,” a Hebrew vocabulary task with “difficult words,” an English vocabulary task with “easy words,” or an English vocabulary task with “difficult words.” As shown in Table 1, there was no difference between the groups in participants’ demographic and language backgrounds.

**Table 1:** Means (and SDs) of participants’ demographic and language background measures by group

	Hebrew easy *N* = 48	Hebrew difficult *N* = 47	English easy *N* = 46	English difficult *N* = 45	*F*	*p*	partial η^2^
**Age**	30.1 (5.7)	29.6 (5.8)	29.0 (5.2)	30.9 (6.0)	.92	.431	.02
**Years of education**	14.2 (2.4)	14.9 (2.4)	14.0 (1.8)	14.9 (1.7)	2.23	.086	.04
**AoA**^**a**^** Hebrew**	.2 (.7)	.2 (.7)	.1 (.6)	.1 (.5)	.22	.885	.00
**AoA**^**a**^** English**	7.0 (4.8)	7.1 (3.0)	7.3 (3.6)	8.2 (5.7)	.66	.580	.01
**SR**^**b**^** Hebrew pre-task**	6.7 (.6)	6.8 (.5)	6.8 (.7)	6.8 (.6)	.17	.918	.00
**SR**^**b**^** Hebrew post-task**	6.6 (.8)	6.5 (.9)	6.7 (.8)	6.7 (.6)	.97	.409	.02
**SR**^**b**^** English pre-task**	4.8 (1.5)	5.0 (1.4)	4.8 (1.4)	5.2 (1.5)	.52	.671	.01
**SR**^**b**^** English post-task**	4.8 (1.5)	4.8 (1.4)	4.8 (1.4)	4.5 (1.4)	.39	.757	.01
**Dominance**^**c**^** pre-task**	1.9 (1.6)	1.9 (1.3)	1.9 (1.6)	1.7 (1.5)	.35	.788	.01
**Dominance**^**c**^** post-task**	1.9 (1.6)	1.6 (1.4)	1.9 (1.5)	2.2 (1.4)	1.07	.361	.02
**Vocabulary score**^**d**^	.9 (.1)	.5 (.3)	.9 (.2)	.4 (.2)	66.62	<.001	.52

^a^ Age of acquisition

^b^ Average self-rated proficiency across speaking, understanding, reading, and writing

^c^ Self-rated proficiency in Hebrew minus self-rated proficiency in English

^d^ Percent correct in the vocabulary task

### Materials

#### Demographic and language history questionnaire

Participants answered demographic questions (e.g., age, gender, years of education) and provided information regarding their Hebrew and English acquisition (e.g., *“At what age did you start learning English?”*). They also rated their speech, comprehension, reading, and writing skills in both Hebrew and English on a scale from 1 (very poor) to 7 (excellent). We informed them that “excellent” referred to performance comparable to that of an educated native speaker. These self-ratings were collected twice, once before the vocabulary tasks (pre-task ratings) and once after (post-task ratings). In the post-task ratings, participants were asked to re-rate their proficiency in both languages and were notified that their responses could be the same as or different from the pre-task ratings, as long as they accurately reflected their proficiency.

#### Construction of vocabulary tasks

To select words for the main task, we conducted two online pretests through the same survey company used to collect the data for the main task (panel4all). In the first pretest, we recruited 58 Hebrew-English bilinguals between the ages of 21 and 40 years (*M* = 29.43, *SD* = 6.06), who were born in Israel and reported no learning disabilities. They acquired English at the mean age of 8.07 years (*SD* = 4.13) and rated their Hebrew proficiency at 6.87 (*SD* = 0.38) and their English proficiency at 4.67 (*SD* = 1.29) on a scale from 1 to 7. Participants completed demographic and language-background questionnaires and then performed the Hebrew and English versions of the Shipley vocabulary task in a counterbalanced order (Hebrew version: Gilboa, unpublished; Mor & Prior, 2020; English version: Shipley, 1940). In each task, they chose the correct meaning of 40 target words from four response alternatives. Accuracy rates were higher in the Hebrew version (*M* =.65, *SD* =.19) than in the English version (*M* =.50, *SD* =.18), *t*(57) = 5.27, *p* <.001, Cohen’s *d* =.69. Because only one item in the English version and six items in the Hebrew version yielded accuracy rates above 85% and could be classified as “easy” items, we created two additional vocabulary tests with a similar structure (selecting the correct meaning of each item from four alternatives), one in Hebrew and one in English, each consisting of 20 relatively familiar words, and ran another pretest.

In the second pretest, 61 Hebrew-English bilinguals who had not participated in the first pretest, aged 20**–**40 years (*M* = 29.14, *SD* = 4.71), were recruited through the same survey company and completed these additional vocabulary tests. All participants were born in Israel. Two reported having learning disabilities and were excluded. The final sample included 59 participants, who on average had acquired English at the age of 7.73 years (*SD* = 1.98) and rated their Hebrew proficiency at 6.87 (*SD* = 0.38) and their English proficiency at 4.88 (*SD* = 1.32). As in the first pretest, accuracy rates were higher for the Hebrew version (*M* =.92, *SD* =.09) than for the English version (*M* =.84, *SD* =.19), *t*(58) = 3.39, *p* =.001, Cohen’s *d* =.44.

Based on these pretests, we selected ten words in each language with accuracy rates above 85% to serve as “easy” words. A paired-samples t-test confirmed that the selected words were matched for difficulty across languages in our sample (*p* =.477). In addition, we selected ten “difficult” words in each language with accuracy rates between 32% and 55%, and verified that these words were also matched for difficulty across languages (*p* =.496). This process resulted in four vocabulary tasks comprising ten “easy” Hebrew words, ten “easy” English words, ten “difficult” Hebrew words, and ten “difficult” English words. Repeated-measures ANOVA on accuracy rates with both language (Hebrew and English) and difficulty level (easy and difficult) as within-item factors showed that there was a main effect of difficulty level, *F*(1, 9) = 1282.03, *MSE* =.00, *p* = <.001, *η*_*p*_^*2*^ =.99, but no main effect of language, *F*(1, 9) = 1.74, *MSE* =.00, *p* =.220, *η*_*p*_^*2*^ =.16, nor an interaction between language and difficulty, *F*(1, 9) =.09, *MSE* =.00, *p* =.768, *η*_*p*_^*2*^ =.01. This analysis confirmed that the tasks in both languages were comparable in both the “easy” and the “difficult” conditions.

#### Vocabulary tasks

Each vocabulary task consisted of ten target words embedded in the phrase (in Hebrew), “Choose the word that has the same meaning as the word….” Participants were presented with the ten words in a randomized order and had to select the correct meaning of each word from four response alternatives with no time limit. The vocabulary score was calculated as the percentage of correct responses to the ten target words.

### Procedure

Participants first answered two screening questions to determine their eligibility for the study (whether they were born in Israel and whether they had any learning disabilities). Then, they completed the pre-task self-ratings in both Hebrew and English. We presented the self-rating questions at the beginning of the questionnaire so that participants would be less likely to remember their exact ratings by the time they reached the post-task ratings. Afterwards, they completed the demographic and language-background questionnaire, performed one of the vocabulary tasks (Hebrew or English, easy or difficult, counterbalanced across participants), and then rated their Hebrew and English proficiency again. The order of languages in the pre- and post-task ratings corresponded to the language of the vocabulary task. For example, participants who completed the vocabulary task in Hebrew rated their Hebrew first and then their English in both the pre- and post-task ratings.

## Results

All analyses were conducted in JASP (JASP Team, 2023). Repeated-measures ANOVA with language (Hebrew and English) and rating time (pre- and post- vocabulary task) as within-subjects variables and group (“easy” Hebrew, “easy” English, “difficult” Hebrew, and “difficult” English) as between-subjects variable predicting self-rated proficiency revealed that participants reported higher proficiency in their L1 (Hebrew; *M* = 6.70, *SD* =.66) than in their L2 (English; *M* = 4.84, *SD* = 1.40), a main effect of language, *F*(1,182) = 308.91, *MS*E = 2.08, *p* <.001, *η*_*p*_^*2*^ =.63. They also reported higher proficiency before the vocabulary task (*M* = 5.86, *SD* =.82) than after it(*M* = 5.68, *SD* =.89), a main effect of time, *F*(1,182) = 33.69, *MSE* =.18, *p* <.001, *η*_*p*_^*2*^ =.16. The interaction between time and group was also significant, *F*(3,182) = 5.32, MSE =.18, *p* =.002, *η*_*p*_^*2*^ =.08, and so was the three-way interaction between language, time, and group, *F*(3,182) = 14.03, MSE =.09, *p* <.001, *η*_*p*_^*2*^ =.19, indicating that the change in self-rating (pre- to post-task change) varied as a function of both language (Hebrew vs. English) and item difficulty (easy vs. difficult). The effect of group, as well as the interactions of language and group, and of language and time, were not significant (*p*s ≥.34).

**Fig. 1:**
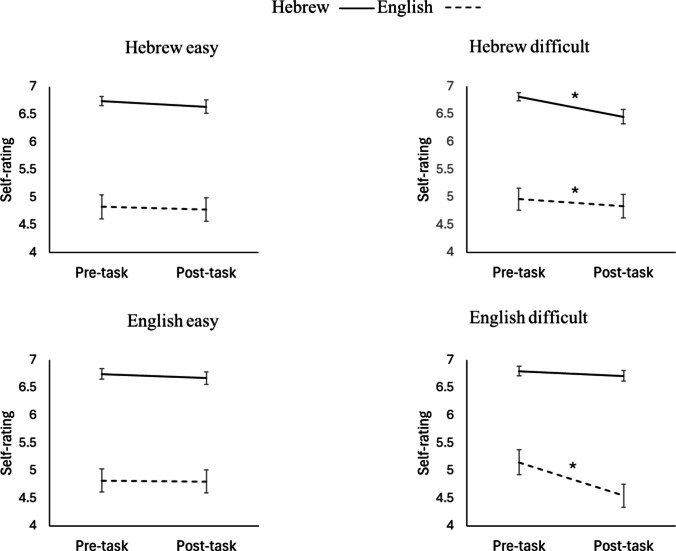
Average (and SE) of pre- and post-task self-ratings of Hebrew and English proficiency across groups

To unpack the three-way interaction, we first examined whether the differences between pre- and post-task ratings in both Hebrew and English were significant within each group separately (see Fig. 1). In the Hebrew easy group, differences were not significant for either Hebrew self-ratings, *t*(47) = 1.73, *p* =.091, Cohen’s *d* =.25, or English self-ratings, *t*(47) = 1.30, *p* =.202, Cohen’s *d* =.19. In the Hebrew difficult group, differences were significant for both Hebrew self-ratings, *t*(46) = 3.78, *p* <.001, Cohen’s *d* =.55, and English self-ratings, *t*(46) = 2.31, *p* =.025, Cohen’s *d* =.34. In the English easy group, differences were not significant for English self-ratings, *t*(45) = 0.14, *p* =.890, Cohen’s *d* =.02, or Hebrew self-ratings, *t*(45) = 1.71, *p* =.095, Cohen’s *d* =.25. Finally, in the English difficult group, differences were significant for English self-ratings, *t*(44) = 5.87, *p* <.001, Cohen’s *d* =.88, but not for Hebrew self-ratings, *t*(44) = 1.91, *p* =.062, Cohen’s *d* =.29.

Since ratings in both Hebrew and English dropped in the Hebrew difficult group, we wanted to examine whether there was an association between the change in both languages in this group (*N* = 47). We found a strong correlation between the change (pre-task minus post-task) in Hebrew and English ratings, (*r* =.62, *p* <.001). A larger drop in Hebrew was associated with a larger drop in English.

Next, we unpacked the three-way interaction of language, time, and group by examining the two-way interaction between group and time separately within each rated language. To examine the magnitude of the difference between pre- and post-task Hebrew self-rating across groups, we conducted a two-way ANOVA with rating time (pre- and post-vocabulary task) as a within-subject variable and group (“easy” Hebrew, “easy” English, “difficult” Hebrew, and “difficult” English) as a between-subject variable, and found the same main effect of rating time, *F*(1, 182) = 23.08, *MSE* =.1, *p* <.001, *η*_*p*_^*2*^ =.11, and an interaction of rating time and group, *F*(3, 182) = 4.35, *MSE* =.1, *p* =.006, *η*_*p*_^*2*^ =.07. The effect of group was not significant, *F*(3, 182) =.27, *MSE* =.88, *p* =.845, *η*_*p*_^*2*^ =.00. Planned contrasts showed that the change in Hebrew self-ratings was larger after completing a difficult vocabulary task in Hebrew than after completing an easy vocabulary task in Hebrew, *t*(182) = 2.80, *p* =.006, Cohen’s *d* =.37, an easy vocabulary task in English, *t*(182) = 3.08, *p* =.002, Cohen’s *d* =.41, and a difficult vocabulary task in English, *t*(182) = 2.92, *p* =.004, Cohen’s *d* =.39 (see Fig. 1). All other comparisons were not significant (*p* ≥.761).

A similar ANOVA comparing the difference between pre- and post-task English self-ratings across groups revealed once more a main effect of rating time, *F*(1, 182) = 22.66, *MSE* =.16, *p* <.001, *η*_*p*_^*2*^ =.11, and an interaction of rating time and group, *F*(3, 182) = 10.58, *MSE* =.16, *p* <.001, *η*_*p*_^*2*^ =.15. The effect of group was not significant, *F*(3, 182) =.04, *MSE* = 3.98, *p* =.988, *η*_*p*_^*2*^ =.00. Planned contrasts showed that the change in English self-ratings was larger after completing a difficult vocabulary task in English than after completing an easy vocabulary task in English, *t*(182) = 4.93, *p* <.001, Cohen’s *d* =.41, an easy vocabulary task in Hebrew, *t*(182) = 4.72, *p* <.001, Cohen’s *d* =.39, and a difficult vocabulary task in Hebrew, *t*(182) = 4.02, *p* <.001, Cohen’s *d* =.33 (see Fig. 1). All other comparisons were not significant (*p* ≥.348).

**Fig. 2:**
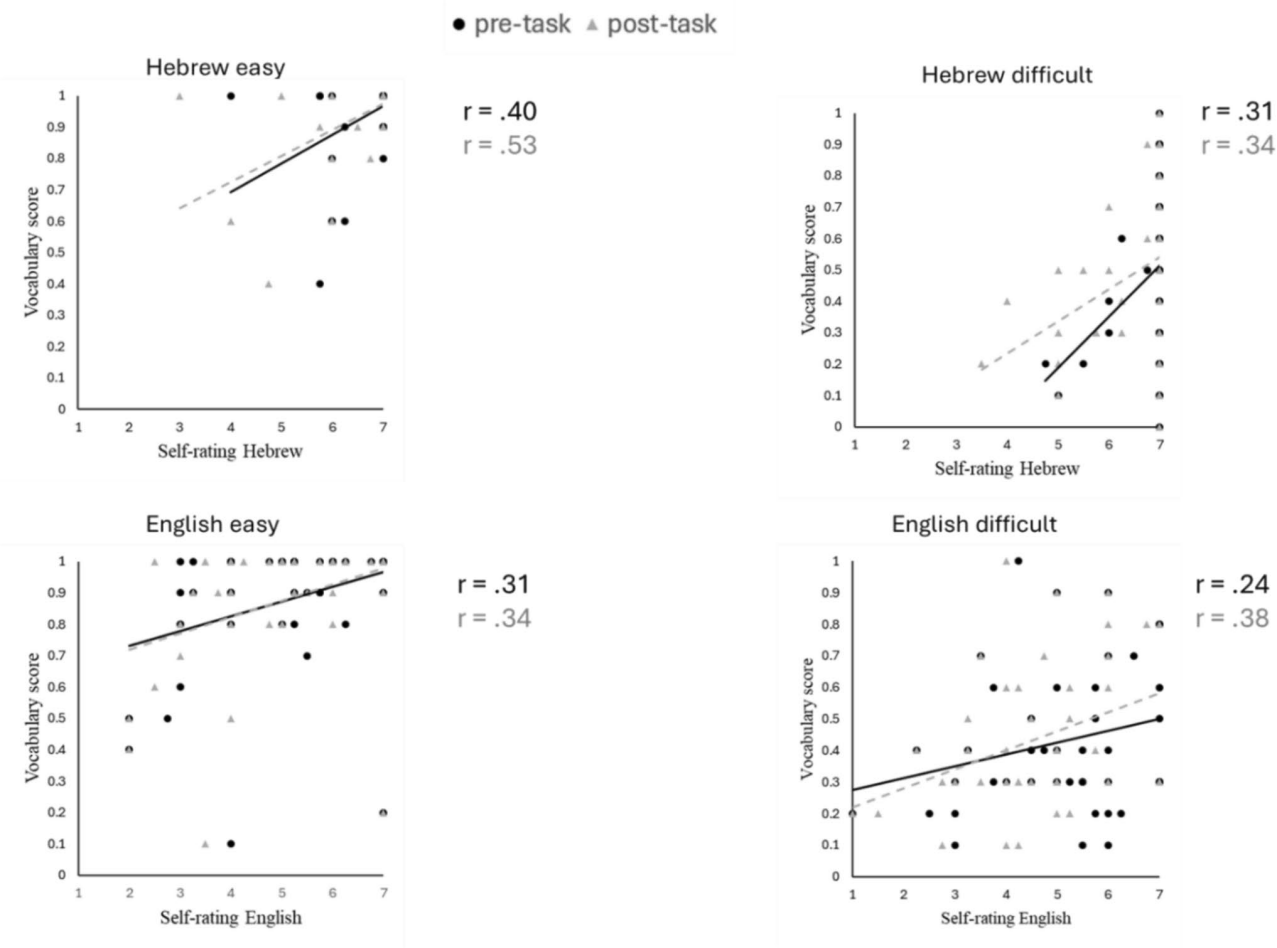
Correlations between pre-and post-task self-ratings and vocabulary scores across groups

To test whether pre- or post-task self-ratings were more indicative of language proficiency, we also examined the correlations between these ratings and vocabulary scores separately for each group (See Fig. 2). In the Hebrew easy group, pre-task self-ratings of Hebrew were correlated with the vocabulary score (*p* =.005), and the correlation was even stronger for the post-task self-ratings (*p* <.001; *z* = 2.23, *p* =.013). In the Hebrew difficult group, pre-task self-ratings of Hebrew were correlated with the vocabulary score (*p* =.032), and the correlation was similar for the post-task self-ratings (*p* =.019; *z* = 0.27, *p* =.392). In the easy English group, pre-task self-ratings of English (*p* =.036) and post-task self-ratings (*p* =.022) were similarly correlated with the vocabulary score (*z* = 0.37, *p* =.357). In the English difficult group, pre-task self-ratings of English were not significantly correlated with the vocabulary score (*p* =.108), but the post-task self-ratings were (*p* =.011; *z* = 2.05, *p* =.002).

## Discussion

Our findings show that self-ratings of proficiency are not fixed measures. Rather, they are dynamic, context-dependent, and shaped by recent experience. After completing a difficult vocabulary task, self-ratings in the language of the task decreased. Moreover, when the task was in the L1, ratings in the L2 decreased as well. Completing an easy vocabulary task, by contrast, did not affect self-ratings in either language. In addition, post-task self-ratings were either as strongly or more strongly correlated with vocabulary scores than pre-task ratings.

In both languages, participants’ self-ratings of proficiency decreased from pre- to post-task when the vocabulary task was difficult, aligning with metacognitive theories, which propose that initial beliefs about one’s knowledge are shaped by and may be revised considering recent experience (Ackerman, 2019; Epstein, 1973; Freund & Kasten, 2012; Koriat, 1997). Pre-task self-ratings likely reflected participants’ general beliefs about their language competence, whereas the post-task ratings reflected a revision of these beliefs following the experience of a challenging task. Previous studies also demonstrated that estimation of self-proficiency may change over time based on experience (Delgado et al., 1999; Dolosic et al., 2016; Ma & Winke, 2019), and show that even a very brief experience can change judgment of self-proficiency.

In the L2, this decrease was not surprising. Initial ratings were already relatively low, and the scale allows for considerable flexibility in interpretation (e.g., the distinction between a 3 and a 4 is not clearly defined). Moreover, Segal and Sidi (2026) found that after completing a difficult L2 vocabulary task, bilinguals became underconfident in their knowledge. The decline in L1 self-ratings is more surprising, as the scale for the L1 was unambiguous, with “7” explicitly defined as “like a native speaker.” In principle, native speakers should remain at this level even when confronted with a difficult task. The observed decrease suggests that participants may not interpret the scale literally but rather subjectively, comparing themselves to an idealized, “perfect” standard. The findings extend those of Tomoschuk and colleagues (2019) and Strong-Krause (2000) by showing that even within the same individuals, interpretations of self-rating scales are context-dependent. They also demonstrate that even strong beliefs about proficiency in L1 may change following experience.

Importantly, although self-ratings in both languages decreased after completing a difficult task in that language, the size of the decrease varied – it was larger in the L2 than in the L1. Delgado and colleagues (1999) also reported a decrease in bilinguals’ self-ratings from pre- to post-task that varied by language. However, their study included only one task in each language, and their participants were relatively balanced bilinguals. Our findings show that the nature of the task is critical for producing this change – the decrease in self-ratings occurred only after a difficult task, and not after an easy one. Moreover, our findings show that the magnitude of the change depends on the initial proficiency, or initial variability, as self-ratings changed less in the more proficient and less variable L1 than the less proficient and more variable L2. These findings suggest that proficiency shapes the momentary experience of difficulty, and that this experience, together with initial beliefs, shapes perceived proficiency.

Following this logic, one might expect that experiencing an easy task, especially in the L2, would increase self-ratings. However, we found no such effect. Self-ratings were similar before and after completing an easy task in both languages. A possible explanation for this is that in both languages, participants were aware of their knowledge of easy words when making the pre-task ratings. Therefore, the experience of success in easy words recognition did not lead to a revision in post-task ratings.

Interestingly, after completing a difficult task in L1, self-ratings decreased not only in L1 but also in L2, and a larger decrease in L1 was associated with a larger decrease in L2. This pattern may imply that bilinguals use their L1 as a reference point against which they evaluate their L2 proficiency. When this reference shifts downward, the perceived proficiency of the L2 shifts as well. This interpretation aligns with the view that self-proficiency judgments are shaped by unique frames of reference that differ for L1 and L2 (Hernandez-Rivera et al., 2024; Tomoschuk et al., 2019). Hernandez-Rivera and colleagues (2024) asked 62 relatively balanced French-English bilinguals (38 French-L1 and 24 English-L1) to rate their proficiency on a 1 to 7 scale and compared these ratings to their performance on a vocabulary task in both languages. They found that participants rated their L1 as maximally proficient regardless of their actual performance, resulting in an overestimation of L1 proficiency (a better-than-average effect; Kruger, 1999). In contrast, they evaluated their L2 by comparing their proficiency to that of native speakers. This comparison with a high-proficiency group led to underestimation of L2 proficiency (a lower-than-average effect). Our findings are consistent with this account and further suggest that the relevant comparison for L2 proficiency may not necessarily be an external group of native speakers, but rather one’s own L1 proficiency.

The findings have methodological, practical, and theoretical implications. Methodologically, researchers often choose the timing of self-ratings within an experimental session based on practical considerations (e.g., reducing fatigue). Some administer them at the beginning of the session (e.g., Garcia & Gollan, 2022; Gollan et al., 2012), whereas others place them at the end (e.g., Hernandez-Rivera et al., 2024; Hirosh & Degani, 2021; Segal & Gollan, 2018). This practice implicitly assumes that the timing of self-ratings does not influence the measure. Our findings indicate that this assumption is unwarranted, as self-ratings are sensitive to immediate experience. Specifically, we found weaker associations between pre-task self-ratings and objective proficiency than between post-task self-ratings and proficiency. Butler and Lee (2006) also found that elementary school students learning English as a foreign language evaluated their English skills more accurately immediately after engaging in an English task than when evaluating their skills more distantly from experience. Note, however, that we do not claim that post-task ratings in our study are inherently more valid, as these effects may be task specific. Nor do we wish to underestimate the potential impact of fatigue on experimental tasks. Rather, the results clearly show that the timing of self-evaluation influences its outcome and should be carefully considered when designing experimental procedures, alongside other methodological factors such as fatigue. This conclusion may apply not only to studies of language, but also to research in other domains in which participants are asked to report on their knowledge, skills, or even emotional states.

Practically, understanding how beliefs and experience shape perceived proficiency is crucial, not only because they can affect the validity of background information collected in bilingualism research, but also because perceived proficiency guides language use and often diverges from actual proficiency (Darasawang & Reinders, 2021; Papi & Khajavy, 2023; Segal & Sidi, 2026). Gaining further insights into how these perceptions are formed may help improve their accuracy and, in turn, influence language use. For example, many L2 users experience linguistic insecurity, defined as a gap between their perceived proficiency and what they conceptualize as standard language knowledge (Labov, 1966; Meyerhoff, 2006). Linguistic insecurity can lead even sufficiently proficient speakers to avoid using the language, limiting practice and enhancing biased self-perceptions (Papi & Khajavy, 2023). Conversely, improving perceived proficiency (e.g., via feedback) can increase confidence and language use. Guerrero-Rodriguez (2025) paired heritage Spanish speakers with fluent Mexican speakers in Mexico for digital interactions and found that most heritage speakers who reported high levels of linguistic insecurity prior to the interaction showed significant increases in confidence by the end of the project, reinforcing the role of experience in shaping perceived proficiency.

Beyond these methodological and practical implications, the findings also carry important theoretical implications. Previous studies have shown that perceived proficiency is shaped by factors such as age (Kavé & Halamish, 2015), emotions (MacIntyre et al., 1997), and social norms (Tomoschuk et al., 2019). Our findings extend this work by demonstrating that the context in which proficiency ratings are made has a substantial effect and can change self-ratings even within the same individuals. In addition, by framing proficiency ratings as context-sensitive metacognitive judgments rather than stable representations of knowledge, we both highlight the indirect relationship between perceived proficiency and actual proficiency and point to the critical role of the interaction between beliefs and recent experience in shaping these judgments (Ackerman, 2019; Koriat, 1997).

Importantly, our findings can advance metacognitive theory, which has largely focused on topics related to memory, comprehension, and reasoning, with relatively little attention given to the assessment of linguistic abilities in general and bilinguals’ language abilities in particular. Our findings suggest that in bilinguals, the assessment of linguistic abilities, especially in L2, is inherently comparative within the self: bilinguals compare their L2 to their L1. Previous studies have suggested that judgments of self-performance in comparative tasks may differ from those in non-comparative tasks (Murphy & Castel, 2024; Segal & Sidi, 2026; Sidi et al., 2020). However, whereas prior comparative studies have typically involved comparisons to others (e.g., judging the originality of one’s own ideas relative to others; Murphy & Castel, 2024; Sidi et al., 2020), comparing judgments of proficiency across languages within the same individual offers a unique and previously unexplored perspective in metacognitive theory.

### Limitations

The study has a few limitations. First, it focused on a specific group of sequential bilinguals, who mostly acquired their L2 through formal education. As such, they received constant feedback on their language ability, probably affecting both their beliefs about their proficiency and their ability to evaluate it. Exploring how language experience influences self-ratings in bilinguals who acquired their L2 differently (e.g., through immersion) will clarify whether the findings generalize to other bilinguals. Second, we chose to focus on a single vocabulary task that is widely used in bilingualism research to manipulate difficulty and feeling of knowing. Testing the effects of a different language experience (e.g., naming pictures) or of feedback (e.g., positive feedback after a difficult item vs. negative feedback after an easy item) will provide a broader understanding of the effect of experience on self-rating. Assessing self-ratings after a delay (e.g., the following day) could also help determine whether the observed changes are transient and tied to the immediate experience, or whether they persist over time. Finally, because the tasks in the easy conditions were intentionally designed to be easy, they inevitably produced higher vocabulary scores and more limited variability than the task in the difficult conditions, which may have affected the correlations observed in the group comparisons. Future studies could address this by using tasks with a wider range of difficulty levels or adaptive tests that maintain variability in performance while still manipulating task experience.

In sum, our findings show that self-rated proficiency is not a stable reflection of language ability, but rather a context-sensitive judgment shaped by both beliefs and recent language experience. The type of experience (easy vs. difficult) and the language in which it occurs shape perceived proficiency. Moreover, difficulty in one’s native language can affect perceived proficiency in the second language, revealing cross-language influences. The findings associate bilingualism research with theories of metacognition and shed light on the processes involved in bilinguals’ estimation of their own proficiency.

## Supplementary Information

Below is the link to the electronic supplementary material.

Supplementary file1 (DOCX 16 KB)

## Data Availability

The datasets generated and/or analyzed during the current study are available via the Open Science Framework (OSF) at: https://osf.io/vk5pt/overview?view_only=d4a3dd220551483e8173be1ccdd29911

## Footnotes

1 Twenty-five participants reported acquiring English before the age of 2 years. We did not exclude them, as all were born in Israel and, except of one participant who reported exposure to Hebrew from the age of 3 years, all were exposed to Hebrew from birth. Nevertheless, we re-analyzed the data without these participants, and the pattern of results remained the same.

## Code availability

The code used to analyze the data is available via the OSF at: https://osf.io/vk5pt/overview?view_only=d4a3dd220551483e8173be1ccdd29911

## Declarations

Research reported in this article complies with institutional ethics requirements that follow American Psychological Association ethical standards. All participants provided informed consent for participation, and the study received institutional ethics approval (3681). Both authors contributed to the study’s conception and design. Material preparation was performed by both authors. Data collection and analysis were conducted by Dorit Segal. The first draft of the manuscript was written by Dorit Segal and Anne Neveu commented on previous versions of the manuscript. Both authors read and approved the final manuscript. The article contains original data that have not been published previously, and it is not currently submitted elsewhere.
