# Supplementary material for: Self-rated language proficiency as a context-dependent judgment
Source: Psychon Bull Rev. 2026 Jun 1;33(5):164. doi: 10.3758/s13423-026-02938-z (PMC13226448; doi:10.3758/s13423-026-02938-z)
Supplement: Supplementary file 1 — Supplementary file1 (DOCX 16 KB) [file 13423_2026_2938_MOESM1_ESM.docx]

**Résumé**

Bien que les auto-évaluations de compétences linguistiques soient largement utilisées dans la recherche sur le bilinguisme et dans le domaine clinique, des études ont montré qu’elles manquent souvent de fiabilité, que ce soit au sein d'une même personne ou bien d'un bilingue, d’une langue ou d’un test à l'autre, ou sur le temps. Pourtant, l’effet potentiel de réaliser ces évaluations en début ou fin de session — et plus spécifiquement, avant ou après un test objectif de compétences — reste méconnu. Nous examinons cette question avec un échantillon de bilingues hébreu-anglais, dominant en hébreu (*N*=186), qui ont auto-évalué leurs compétences dans les deux langues avant et après avoir passé un test de vocabulaire à choix multiple dans l’une des deux langues. De manière cruciale, nous avons manipulé la difficulté du test (niveau facile ou difficile) pour mieux comprendre le rôle du contexte dans l’auto-évaluation des compétences linguistiques. Les résultats montrent que les auto-évaluations sont influencées par l’expérience récente : quelle que soit la langue, lorsque le test de vocabulaire était difficile, les scores des auto-évaluations dans la langue du test ont diminué. De plus, lorsque la version difficile du test était dans la langue maternelle, les auto-évaluations dans la seconde langue (qui n’était pas testée) ont également diminué. Enfin, les auto-évaluations post-test tendaient à être plus fortement corrélées avec les scores de vocabulaire que les évaluations pré-test. Dans l’ensemble, ces résultats montrent que les auto-évaluations de compétences linguistiques sont des inférences dynamiques et dépendantes du contexte, façonnées à la fois par des croyances préexistantes sur les compétences linguistiques et par l’expérience immédiate, soulignant ainsi la nécessité de prendre en compte les facteurs individuels et liés aux tests qui peuvent influencer les auto-évaluations lors de la collecte de données.

Mots clés : Bilinguisme, Langue étrangère, Auto-évaluations des compétences linguistiques

**Resumen**

Aunque las autoevaluaciones de la competencia lingüística se utilizan ampliamente en la investigación sobre el bilingüismo y en el ámbito clínico, diversos estudios han demostrado que suelen carecer de fiabilidad, ya sea a nivel intraindividual o interindividual, entre lenguas, tareas o a lo largo del tiempo. Sin embargo, el efecto potencial de realizar estas evaluaciones al principio o al final de una sesión de evaluación —y, más específicamente, antes o después de una prueba objetiva de competencia— sigue siendo poco conocido. Examinamos esta cuestión en una muestra de bilingües hebreo-inglés con dominancia en hebreo (*N*=186), quienes autoevaluaron sus competencias en ambas lenguas antes y después de realizar una prueba de vocabulario de opción múltiple en uno de los dos idiomas. De manera crucial, manipulamos la dificultad de la prueba (nivel fácil o difícil) para comprender mejor el papel del contexto en la autoevaluación de la competencia lingüística. Los resultados muestran que las autoevaluaciones se ven influenciadas por la experiencia reciente: independientemente de la lengua, cuando la prueba de vocabulario era difícil, las puntuaciones de las autoevaluaciones en la lengua de la prueba disminuyeron. Además, cuando la versión difícil de la prueba se realizó en la lengua materna, las autoevaluaciones en la segunda lengua (que no estaba siendo evaluada) también disminuyeron. Por último, las autoevaluaciones posprueba tendieron a correlacionar más fuertemente con las puntuaciones de vocabulario que las evaluaciones preprueba. En conjunto, estos resultados demuestran que las autoevaluaciones de la competencia lingüística son inferencias dinámicas y dependientes del contexto, modeladas tanto por creencias preexistentes sobre las habilidades lingüísticas como por la experiencia inmediata, lo que subraya la necesidad de considerar los factores individuales y los relacionados con las pruebas que pueden influir en las autoevaluaciones durante la recogida de datos.

**Palabras clave:** Bilingüismo; Lengua extranjera; Autoevaluación de la competencia lingüística
